# An integrated censored and uncensored (ICU) twin model for biomarker heritability estimation

**DOI:** 10.1002/alz70856_105865

**Published:** 2026-01-08

**Authors:** Nathan A. Gillespie, Michael C. Neale, Luis F S Castro‐de‐Araujo, James Barlow, Robert A. Rissman, Tina T. Vo, Tyler R. Bell, Chandra A. Reynolds, Carol E. Franz, William S. Kremen, Matthew S. Panizzon, Jeremy A. Elman, Vietnam Era Twin Study of Aging (VETSA)

**Affiliations:** ^1^ Virginia Commonwealth Univeristy, Richmond, VA, USA; ^2^ QIMR Berghofer Medical Research Institute, Brisbane, QLD, Australia; ^3^ The University of Melbourne, Melbourne, VIC, Australia; ^4^ Virginia Commonwealth University, Richmond, VA, USA; ^5^ University of Southern California, San Diego, CA, USA; ^6^ Alzheimer's Therapeutic Research Institute, University of Southern California, San Diego, CA, USA; ^7^ University of California San Diego, La Jolla, CA, USA; ^8^ University of Colorado Boulder, Boulder, CO, USA; ^9^ Center for Behavior Genetics of Aging, University of California, San Diego, La Jolla, CA, USA

## Abstract

**Background:**

Plasma phosphorylated tau at threonine 231 (pTau231) is a promising Alzheimer's disease biomarker, especially for preclinical stages, though early measurements often fall below detection limits. Assay detection limits create left‐censored data that can bias biomarker heritability estimates and associations with other variables. Standard approaches such as treating values as continuous, setting censored values to zero, or coding them as missing can bias results. We developed a model to estimate heritability that integrates censored and uncensored observations, providing a framework for analyzing left‐censored biomarker data.

**Method:**

We analyzed plasma pTau231 data from Quanterix Simoa assays of 1,071 male twins aged 60‐73 years within the Vietnam Era Twin Study of Aging. Our Integrated Censored and Uncensored (ICU) model treats observations below the limit of detection (LOD=0.621 pg/mL) as ordinal data and above‐limit values as continuous measures before decomposing variance into additive genetic effects, shared environmental factors common to twins, and unique environmental influences specific to each twin.

**Result:**

Using our ICU model adjusted for site and storage time covariates, we found that plasma pTau231 was moderately heritable, with additive genetic influences explaining 38% of the variance (95% CI: 0.26‐0.48), while unique environmental influences explained the remaining 62%. Different approaches to handling censored data produced varying results, with some methods suggesting no genetic influence. Simulation studies will demonstrate conditions where the ICU maintains reliable performance while other approaches may produce biased estimates.

**Conclusion:**

Our ICU modeling strategy offers a theoretically grounded approach for estimating heritability from left‐censored biomarker data without requiring arbitrary decisions about censored values. These findings have implications for genetic association studies and other biomarker analyses ‐ appropriate handling of censored values is crucial since different approaches can substantially alter estimates of genetic influence, potentially leading to spurious or missed genetic associations. The moderate heritability of plasma pTau231 supports its potential as an AD biomarker while suggesting substantial environmental influence on individual differences.

**Reference**

1. Snieder, H., Boomsma, D.I., Van Doornen, L.J., Neale, M.C. (1999) Bivariate genetic analysis of fasting insulin and glucose levels. *Genet Epidemiol*. 16:426‐446.

